# Acute Antiplatelet Effects of an Oleocanthal-Rich Olive Oil in Type II Diabetic Patients: A Postprandial Study

**DOI:** 10.3390/ijms25020908

**Published:** 2024-01-11

**Authors:** Maria Efthymia Katsa, Kleopatra Ketselidi, Marianna Kalliostra, Anastasios Ioannidis, Andrea Paola Rojas Gil, Panagiotis Diamantakos, Eleni Melliou, Prokopios Magiatis, Tzortzis Nomikos

**Affiliations:** 1 Department of Nutrition and Dietetics, School of Health Sciences and Education, Harokopio University of Athens, GR-17676 Athens, Greece; merfykat@hua.gr (M.E.K.); kleokets@gmail.com (K.K.); marianna.kalliostra@efad.org (M.K.); 2 Laboratory of Biology and Biochemistry, Department of Nursing, Faculty of Health Sciences, University of Peloponnese, GR-22100 Tripoli, Greece; tasobi@uop.gr (A.I.); arojas@uop.gr (A.P.R.G.); 3 Laboratory of Pharmacognosy and Natural Products Chemistry, Department of Pharmacy, National and Kapodistrian University of Athens, GR-15774 Athens, Greece; pdiam@pharm.uoa.gr (P.D.); emelliou@pharm.uoa.gr (E.M.); magiatis@pharm.uoa.gr (P.M.)

**Keywords:** oleocanthal-rich olive oil, oleocanthal, type 2 diabetes mellitus, platelet aggregation, postprandial, dysmetabolism

## Abstract

Postprandial dysmetabolism is a common entity of type 2 diabetes mellitus (T2DM) and may act as a daily stressor of the already dysfunctional diabetic platelets. This study aims to investigate whether oleocanthal-rich olive oils (OO), incorporated into a carbohydrate-rich meal, can affect postprandial dysmetabolism and platelet aggregation. Oleocanthal is a cyclooxygenase inhibitor with putative antiplatelet properties. In this randomized, single-blinded, crossover study, ten T2DM patients consumed five isocaloric meals containing 120 g white bread combined with: (i) 39 g butter, (ii) 39 g butter and 400 mg ibuprofen, (iii) 40 mL OO (phenolic content < 10 mg/Kg), (iv) 40 mL OO with 250 mg/Kg oleocanthal and (v) 40 mL OO with 500 mg/Kg oleocanthal. Metabolic markers along with ex vivo ADP- and thrombin receptor-activating peptide (TRAP)-induced platelet aggregation were measured before and for 4 h after the meals. The glycemic and lipidemic response was similar between meals. However, a sustained (90–240 min) dose-dependent reduction in platelets’ sensitivity to both ADP (50–100%) and TRAP (20–50%) was observed after the oleocanthal meals in comparison to OO or butter meals. The antiplatelet effect of the OO containing 500 mg/Kg oleocanthal was comparable to that of the ibuprofen meal. In conclusion, the consumption of meals containing oleocanthal-rich OO can reduce platelet activity during the postprandial period, irrespective of postprandial hyperglycemia and lipidemia.

## 1. Introduction

Postprandial dysmetabolism is characterized by hyperglycemia and hypertriglyceridemia along with the increased production of atherogenic apoB lipoproteins after the consumption of energy dense meals rich in carbohydrates and fat [1]. Hyperglycemia and hypertriglyceridemia may in turn trigger subclinical activation of inflammation and oxidative stress. The aforementioned conditions are driven by insulin resistance and the inability of the homeostatic systems to effectively metabolize macronutrients [2]. Therefore, postprandial inflammation and oxidative stress are common features of obesity, metabolic syndrome and Type II diabetes (T2DM), making postprandial dysmetabolism a chronic stressor for the cardiometabolic system of those patients [2,3].

Although the majority of postprandial studies have focused on the implications of postprandial dysmetabolism on inflammation, oxidative stress and endothelial dysfunction, it seems that postprandial dysmetabolism can also induce transient alterations of the hemostatic system [4,5]. Platelets are sensitive responders of the postprandial alterations since both oxidative stress and inflammatory mediators can activate them [6,7]. Diabetic platelets seem to be more prone to activation under hyperglycemic, hyperinsulinemic and hypertriglyceridemic conditions, taking into account that they are more sensitive to endogenous platelet activators [8]. The diabetic platelets have various dysregulated signaling cascades causing platelet activation. Specifically, they have increased amounts of intracellular calcium [9], elevated microparticles, increased glycoprotein and P-selectin expression, decreased membrane fluidity and lower cyclic adenosine monophosphate (cAMP) amounts leading to increased G protein-coupled purinergic receptor (P2Y12) signaling. Moreover, diabetic platelets show short-term calcium-sensitive protein kinase C (PKC) β isoenzyme’s activation [8,10,11] during an event of acute hyperglycemia in vitro. It is therefore obvious that the postprandial dysmetabolism of diabetic patients may be a chronic stimulus of platelet activation, leading to the release of pro-inflammatory and pro-atherogenic mediators from their granules. 

The macronutrient and micronutrient content of the meals may have a strong impact, either positive or negative, to all features of postprandial dysmetabolism, including platelet activation [3,12]. The recent literature has highlighted the beneficial effects of extra virgin olive oil (EVOO) minor components on atherosclerosis and thrombotic risk [13,14]. Among other microconstituents, EVOO contains at least 40 phenolic compounds, classified as phenolic acids, phenolic alcohols, secoiridoids, flavonoids, lignans and hydroxy-isochromanes. According to in vitro and preclinical studies, EVOO phenolics reduce coagulation, endothelial dysfunction, inflammation and oxidative stress, pathological mechanisms’ responsible for the majority of chronic diseases [15,16,17]. The polyphenols of EVOO have demonstrated antiplatelet properties, mainly in vitro [15]. However, the exact mechanisms of these actions in vivo have not been demonstrated yet, as the phenolic profile of EVOOs used in intervention studies are rarely characterized [15]. Oleocanthal is the most promising antiplatelet component of EVOO, mainly due to its ability to inhibit the cyclooxygenase (COX) activity and the subsequent production of thromboxane A2 [15,18,19]. Its anti-inflammatory properties resemble that of ibuprofen in terms of both potency and action [18]. The acute antiplatelet properties of an olive oil rich in oleocanthal and oleacein have already been demonstrated in healthy subjects [20]. Whether those properties remain the same in the context of a carbohydrate meal in type II diabetic patients is not known. Therefore, the aim of this study is to compare the metabolic and platelet responses of diabetic patients to five meals consisting of white bread along with either butter (BU), butter and ibuprofen (BU-IBU), olive oil with low phenolic content (OO) and olive oil containing 250 mg/Kg oleocanthal (OO250) and 500 mg/Kg oleocanthal (OO500) without other detectable phenolics. This set of meals allows several comparisons between saturated fatty acid and monounsaturated fatty acid rich meals (BU vs. OO), between meals with olive oils both poor and rich in oleocanthal (OO vs. OO250 and OO500) and between the administration of ibuprofen (as a positive control) and oleocanthal (BU-IBU vs. OO250 and OO500). 

## 2. Results

### 2.1. Baseline Characteristics

Ten volunteers with T2DM (5 females) completed the protocol. No differences were observed between baseline anthropometric, clinical and biochemical markers prior to each intervention. Analysis of the 24 h dietary recalls revealed no significant differences for the intake of energy and macronutrients before each intervention (Table 1 and Table 2).

### 2.2. Effect of Meals Consumption on Glycemic Response

**Figure 1:**
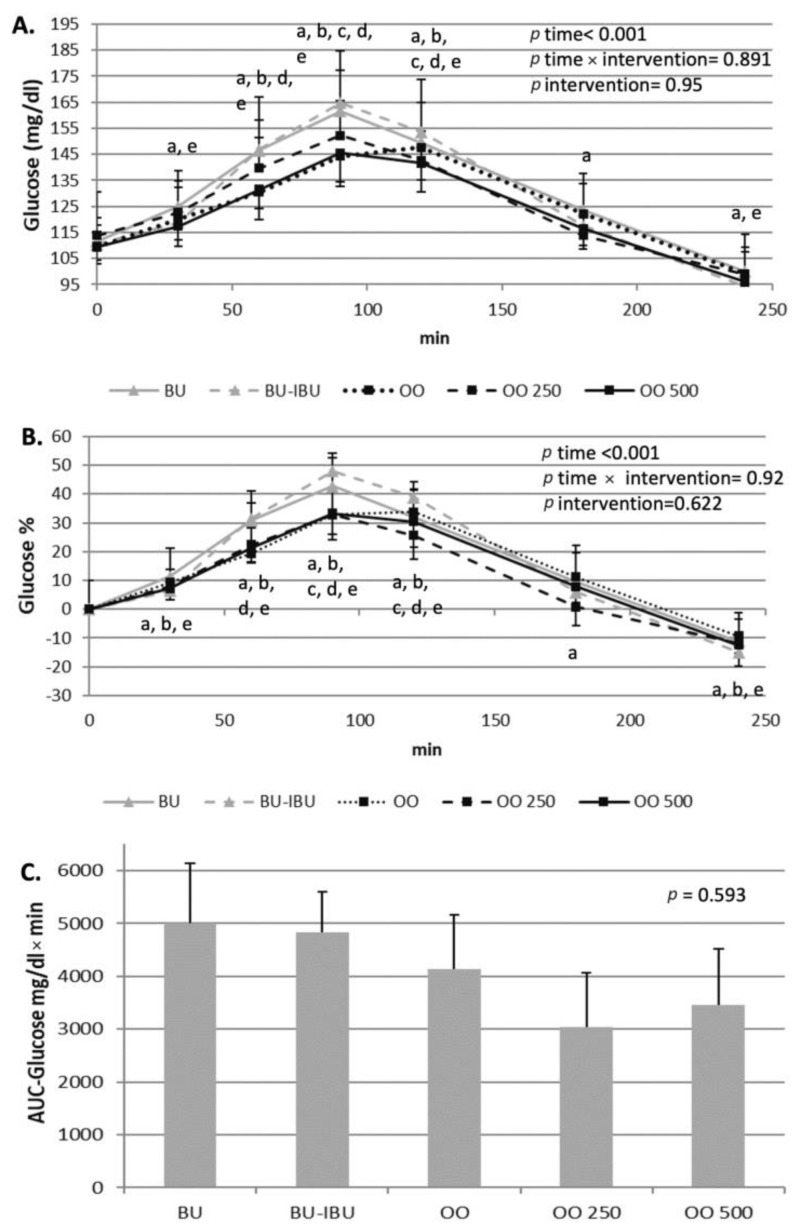
Effect of meals consumption on the glucose response. (**A**) Absolute values, (**B**) % changes and (**C**) iAUCs for the response of glucose after each meal. Data were analyzed with ANOVA for repeated measurements, followed by a Bonferroni test for specific time points. Significant differences between post-meal vs. pre-meal values (*p* < 0.05) are depicted with the letters a: BU, b: BU-IBU, c: OO, d: OO250 and e: OO500.

**Figure 2:**
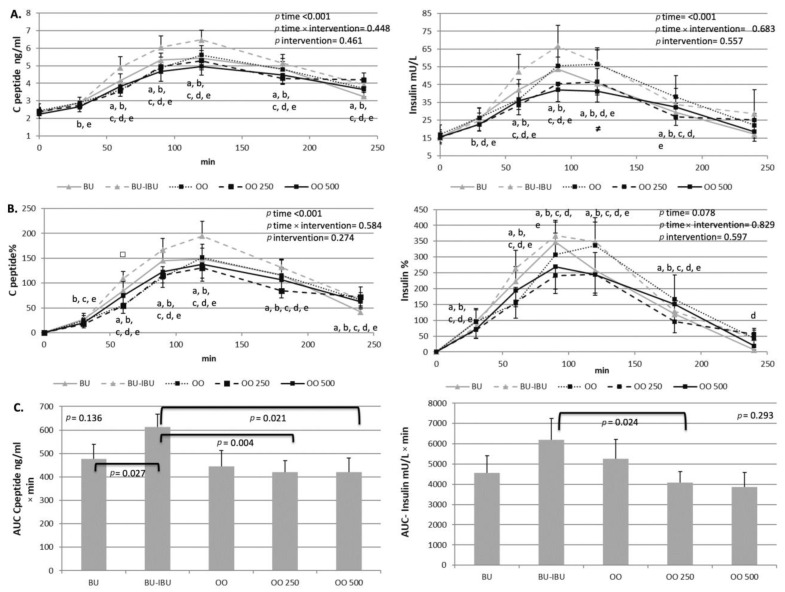
Effect of meals consumption on the C-peptide and insulin response. (**A**) Absolute values, (**B**) % changes and (**C**) iAUCs for the response of C-peptide and insulin after each meal. Data were analyzed with ANOVA for repeated measurements, followed by a Bonferroni test for specific time points. Significant differences between post-meal vs. pre-meal values (*p* < 0.05) are depicted with the letters a: BU, b: BU-IBU, c: OO, d: OO250 and e: OO500. Significant difference (*p* < 0.05) between the studied groups at the same time point is depicted with the symbols→ ≠: OO vs. OO250, □: OO250 vs. OO500.

All meals were able to increase glucose levels, with a peak observed at t = 90–120 min. According to the glucose’s incremental area under the curve (iAUC) values, no differences were observed between meals, although there is a non-significant trend for lower AUC values after the OO250 and OO500 meals compared to the other meals. A slight hypoglycemia was observed at t = 240 min with an approximately 10% lower glucose concentration compared to the baseline values, specifically after the BU and OO500 interventions. A transient increase of insulin and C-peptide was observed after the consumption of all meals, with a peak at 90 min or 120 min depending on the consumed meal. The intake of ibuprofen seems to induce a higher response to insulin compared to other meals; however, statistical significance was observed only for the insulin’s iAUC between BU-IBU and OO250. A similar trend was observed for the C-peptide responses where C-peptide’s iAUC after BU-IBU was significantly higher compared to BU, OO250, and OO500 (Figure 1 and Figure 2).

### 2.3. Effect of Meals Consumption on Lipid Profile

**Figure 3:**
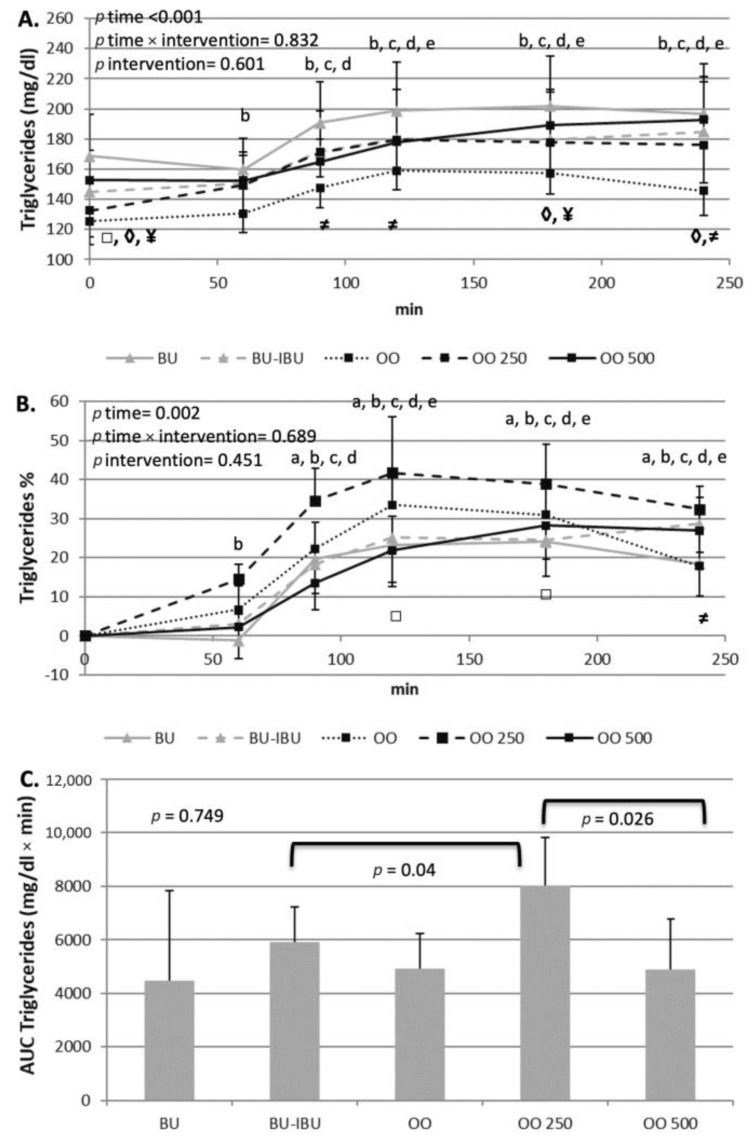
Effect of meals consumption on the TGs response. (**A**) Absolute values, (**B**) % changes and (**C**) iAUCs for the response of TGs after each meal. Data were analyzed with ANOVA for repeated measurements, followed by a Bonferroni test for specific time points. Significant differences between post-meal vs. pre-meal values (*p* < 0.05) are depicted with the letters a: BU, b: BU-IBU, c: OO, d: OO250 and e: OO500. Significant differences (*p* < 0.05) between two studied groups at the same time point is depicted with the symbols→ ¥: OO vs. BU, ≠: OO vs. OO250, ◊: OO vs. OO500, □: OO250 vs. OO500.

**Figure 4:**
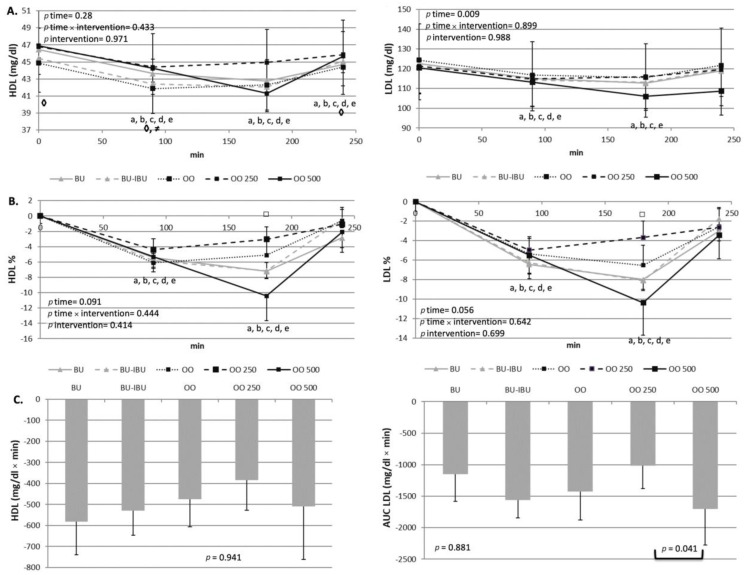
Effect of meals consumption on the HDL-C and LDL-C response. (**A**) Absolute values, (**B**) % changes and (**C**) iAUCs for the response of LDL-C and HDL-C after each meal. Data were analyzed with ANOVA for repeated measurements, followed by a Bonferroni test for specific time points. Significant differences between post-meal vs. pre-meal values (*p* < 0.05) are depicted with the letters a: BU, b: BU-IBU, c: OO, d: OO250 and e: OO500. Significant difference (*p* < 0.05) between studied groups at the same time point is depicted with the symbols→ ≠: OO vs. OO250, ◊: OO vs. OO500, □: OO250 vs. OO500.

As expected, an increase in the level of triglycerides (TGs) was observed after the consumption of all meals. TG increments started one hour after the meals’ consumption. A broad peak between 120 and 240 min is observed. The TG levels did not return to baseline values until 4 h postprandially. A higher TG response was observed after the consumption of OO250, which reached statistical significance only when it was compared with BU-IBU and OO500. A small but significant decrease in LDL-C levels was observed after the consumption of all meals. A broad peak was observed between 90 to 180 min showing decrements of 4–10%. No significant differences amongst LDL-C iAUCs were observed, with the exception of the iAUC of OO250 which was significantly lower than the iAUC of OO500. A similar trend was shown for HDL-C. However, no significant differences were observed between meals (Figure 3 and Figure 4).

### 2.4. Effect of Interventions on Uric Acid and Homocysteine

A small but significant decline of uric acid was observed 3 h after the consumption of all meals (4–7%). The magnitude of the decline was similar for all meals. The levels of uric acid did not return to baseline values after the BU, OO and OO250 meals. A transient, albeit non-significant, reduction in homocysteine was observed after the consumption of all meals at 90 min. No differences on the kinetics of homocysteine were observed between meals (Figures S1 and S2).

### 2.5. Effect of Interventions on Platelet Activity Indices

**Figure 5:**
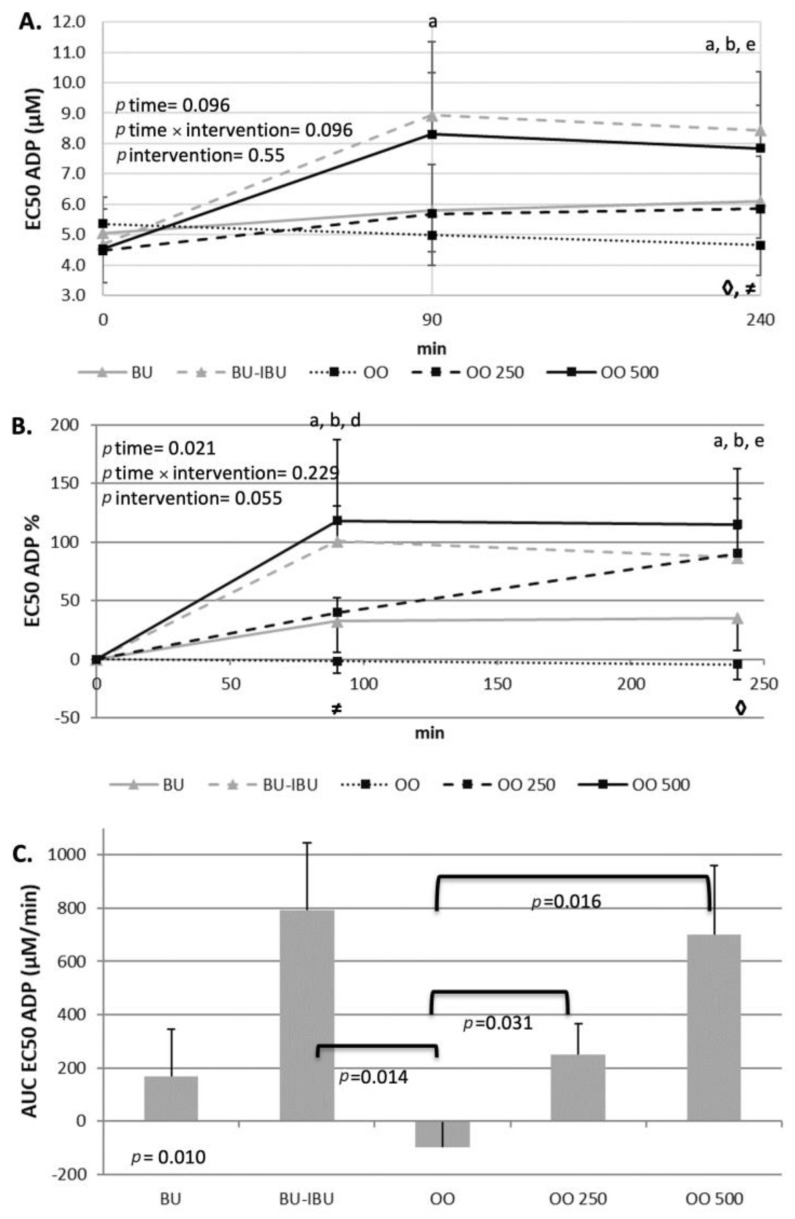
Effect of meal consumption on EC_50_ values of ADP-induced platelet aggregation. (**A**) Absolute values, (**B**) % changes and (**C**) iAUCs for the response of platelets after each meal, using the aggregating factor ADP. Data were analyzed with ANOVA for repeated measurements, followed by a Bonferroni test for specific time points. Significant differences between post-meal vs. pre-meal values (*p* < 0.05) are depicted with the letters a: BU, b: BU-IBU, d: OO250 and e: OO500. Significant difference (*p* < 0.05) between two studied groups at the same time point is depicted with the symbols→ ≠: OO-OO250, ◊: OO-OO500.

**Figure 6:**
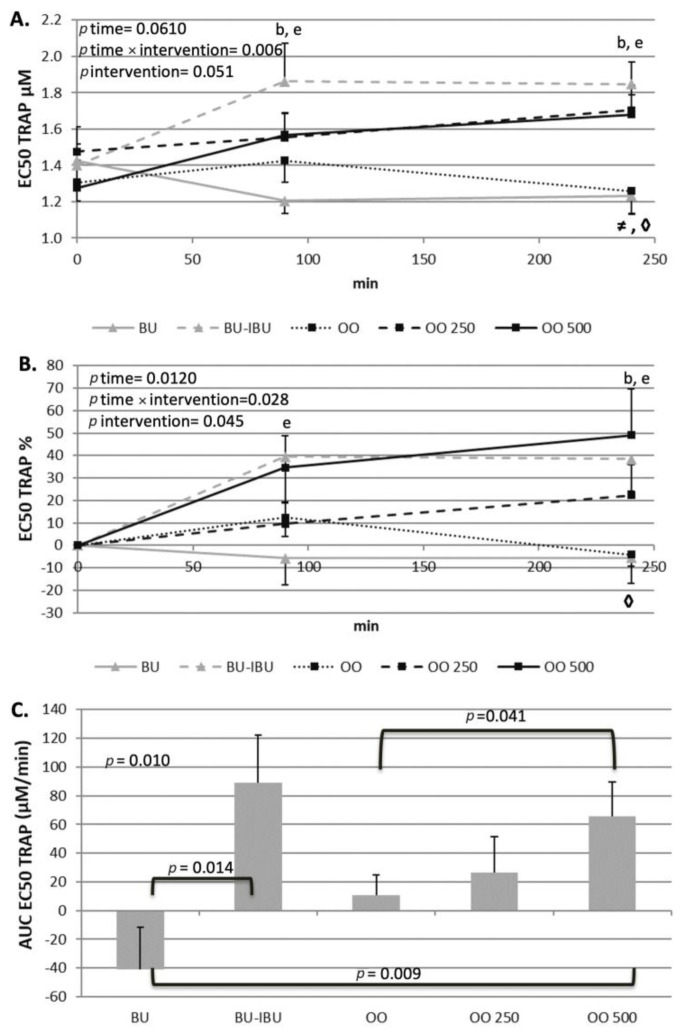
Effect of meals consumption on EC_50_ values of TRAP-induced platelet aggregation. (**A**) Absolute values, (**B**) % changes and (**C**) iAUCs for the response of platelets after each meal, using the aggregating factor TRAP. Data were analyzed with ANOVA for repeated measurements, followed by a Bonferroni test for specific time points. Significant differences between post-meal vs. pre-meal values (*p* < 0.05) are depicted with the letters, b: BU-IBU, and e: OO500. Significant difference (*p* < 0.05) between two studied groups at the same time point is depicted with the symbols→ ≠: OO vs. OO250, ◊: OO vs. OO500.

**Figure 7:**
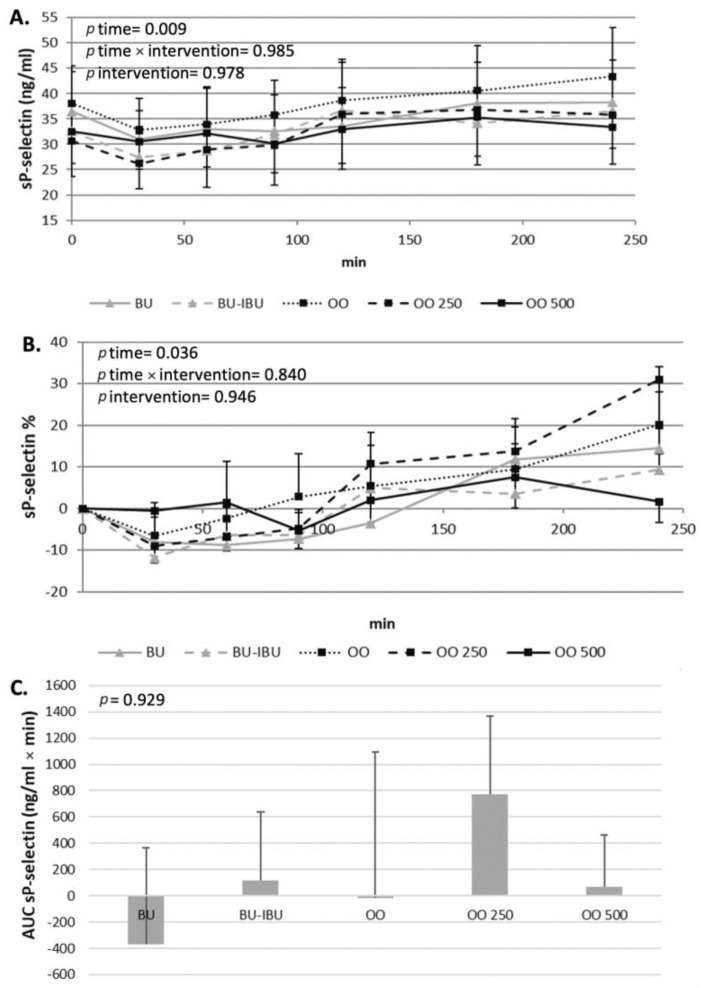
Effect of meals consumption on sP-selectin. (**A**) Absolute values, (**B**) % changes and (**C**) iAUCs for the response of sP-selectin after each meal. Data were analyzed with ANOVA for repeated measurements, followed by a Bonferroni test for specific time points.

The response of participants’ platelets after the consumption of each meal depended on both the type of meal and the aggregating factor. An increase in EC_50_ values implies the desensitization of platelets to the aggregating agent while a lowering of EC_50_ values implies that the platelets are more prone to activation. A neutral response of platelets to both activating factors was observed after the consumption of OO. The EC_50_ values remained the same throughout the four-hour postprandial period. In contrast to this response, a dose-dependent ex vivo deactivation of platelets was observed after the consumption of OO250 and OO500. The consumption of BU resulted in a slight, significant and sustained increase in EC_50_ values when adenosine 5′-diphosphate (ADP) was the aggregating agent. A different response of platelets to BU was observed when the aggregating agent was thrombin receptor-activating peptide (TRAP) since a non-significant reduction in EC_50_ values was shown. As expected, BU-IBU increased the EC_50_ of platelets to both aggregating factors; however, only after TRAP activation was this increase significantly higher compared to BU meal. It should be mentioned that the OO500 meal had a similar potential to attenuate platelet activation with that of BU-IBU. Concerning sP-selectin, its concentration did not change significantly after all meals, although a trend for elevated levels at 4 h was observed (Figure 5, Figure 6 and Figure 7). 

## 3. Discussion

T2DM is a hypercoagulable state characterized by dysfunctional platelets. The diabetic platelet shows increased P2Y12 signaling, oxidative stress, P-selectin and glycoprotein expression along with resistance to antiplatelet therapy, decreased membrane fluidity and increased intracellular Ca^2+^ [11,15]. A chronically dysfunctional platelet is able to initiate and propagate cellular mechanisms which lead to atherothrombosis and vascular inflammation, hallmarks of the diabetic complications. Most dietary interventions on T2DM patients are mainly focused on hyperglycemia, hyperlipidemia and subclinical inflammation, and there are not many studies focusing on the dysregulated hemostatic systems and the dysfunctional diabetic platelets [21]. The postprandial period is a daily stressor of the metabolic homeostasis and it seems that, under conditions of insulin resistance, postprandial hyperglycemia and hyperlipidemia can induce a transient activation of platelets [22,23,24]. On the other hand, it is not known whether a putative postprandial activation of platelets can be attenuated if the meals include foods or mixtures of micronutrients/phytochemicals with antiplatelet properties. The antiplatelet actions of olive oil phenolics are mainly proven in cellular experiments [15]. Amongst all olive oil polyphenols, oleocanthal is a potent inhibitor of cyclooxygenase COX1, which in turn mediates the biosynthesis of TxA2, a strong endogenous platelet agonist [19]. The consumption of olive oils rich in oleocanthal by healthy volunteers acutely demonstrates ex vivo antiplatelet properties [20]. However, it is not known whether the diabetic platelets can respond similarly to oleocanthal-rich OOs. Our results have shown that the consumption of an olive oil rich in oleocanthal (250 and 500 mg/Κg) by T2DM patients can acutely and in a dose-dependent manner reduce the sensitivity of platelets to ADP- and TRAP-induced ex vivo platelet aggregation without affecting the postprandial response of metabolic markers. 

The current study was conducted through a crossover design, as T2DM patients show a wide heterogeneity regarding their clinical profile and medication received, which may affect their postprandial response. All test meals consisted of white bread as a source of glucose. The amount of glucose in the test meals (50 g) is sufficient to induce a significant postprandial hyperglycemia and hyperinsulinemia to T2D patients. It should be mentioned that the dipping of bread in olive oil is a common way by which Greeks and other Mediterranean populations consume olive oil. In order to assess the ability of oleocanthal-rich olive oils to affect postprandial dysmetabolism and platelet activation, we used two olive oils with different enrichment in oleocanthal, specifically 250 and 500 mg/Kg. Olive oils with similar enrichments in oleocanthal, when provided to healthy volunteers at the same amount as in our study (40 mL), showed a strong acute, ex vivo, antiplatelet effect [20]. We administered olive oils with a different enrichment in oleocanthal to assess whether the postprandial response of volunteers is dose-dependent. The control olive oil (OO) had the same fatty acid profile as the enriched olive oils since they are all derived from the same oleocanthal-rich olive oil. The BU meal was included in the study so we could compare the response of the diabetic patients to a saturated fatty acid (SFA) and a monounsaturated fatty acid (MUFA) meal and the impact of MUFA on postprandial metabolism. Finally, the BU-IBU meal played the role of the positive control meal in terms of its antiplatelet properties. In order to achieve a similar biochemical and nutritional profile of our volunteers between trials, dietary instructions were given before the interventions, which were similar to those given before the oral glucose tolerance test (OGTT) [25]. Additionally, the volunteers were advised to avoid polyphenol-rich food and foods rich in antiplatelet constituents for three consecutive days before trials [26]. Table 1 clearly shows that the dietary intake of macro- and micronutrients was similar between trials. The selection of the biochemical indices that were finally measured in this study was based on their ability to affect platelet functionality and reflect postprandial dysmetabolism. 

The present study showed that all meals increased glucose, insulin and C-peptide levels postprandially. Although RM-ANOVA analysis did not demonstrate a time x meal or meal effect on the kinetics of glycemic response markers, pairwise comparisons between the respective iAUCs have shown a trend for a higher insulin and C-peptide response after the BU-IBU compared to the other meals. It is therefore obvious that neither the type of fat source nor the enrichment of olive oil with oleocanthal can have a strong impact on postprandial glycemia and insulinemia. The effect of fatty acid composition of the meal on the acute glycemic response of T2D patients has not been studied thoroughly and the few studies that have been conducted show contradictory results when they compare SFA-rich with MUFA-rich meals [27,28,29]. The similar glycemic and insulinemic responses after the consumption of oleocanthal-poor and oleocanthal-rich meals imply that the enrichment of olive oil with oleocanthal did not confer additional benefit compared to olive oils poor in oleocanthal. Previous studies have shown that EVOOs, compared to OOs, can lower postprandial hyperglycemia in patients with impaired fasting glucose [30], non-obese patients and those with morbid obesity after Roux-en-Y gastric bypass [31]. This effect was accompanied by higher insulin responses mediated by the ability of EVOOs to augment the secretion of incretins. In our study, such an effect was not observed, either because of the long history of diabetes in our patients or because a milieu of olive oil phenolics is required for the improvement of the glycemic response compared to oleocanthal alone. Finally, an interesting finding of our study is the ability of ibuprofen to induce a higher response of insulin and C-peptide compared to other meals. Although this effect has not been proven in other postprandial studies, animal studies have shown the ability of non-steroidal anti-inflammatory drugs to increase insulin release from beta cells by inhibiting ATP-sensitive potassium channels [32]. Additionally, the NSAIDs’ antidiabetic role may be correlated with the reduced insulin clearance [33]. Experiments conducted in mice models noted naproxen’s ability to reduce glucose and increase liver’s glycogen and serum insulin, C-peptide and adiponectin [33,34].

All meals were able to induce mild postprandial increases in TG levels, starting at 90 min and lasting up to 240 min when TG levels did not return to baseline values. No time x meal or meal effect was observed, but pairwise comparison between iAUCs demonstrated a dose-dependent effect of oleocanthal. Specifically, a higher TG response to OO250 compared to OO500 and OO (trend) was observed. Previous studies have shown that EVOO was able to increase GLP1 release after a meal in insulin-resistant patients [30]. GLP1 is capable of slowing the gastric emptying or down-regulating the Apo B-48 intestinal tube secretion, which is responsible for chylomicrons transport in the lymphatic and systemic circulation [35]. Whether oleocanthal-rich olive oils act similarly to EVOO is not known. It is also unclear why OO250 induced a higher postprandial hypertriglyceridemia compared to OO500. Since both olive oils induced similar increases of insulin and C-peptide, we cannot attribute this dose effect on the insulin-driven metabolism of TGs, but probably to intraluminal mechanisms of TG absorption. 

LDL-C and HDL-C postprandial kinetics showed a similar pattern. A transient decrease was observed between 90–180 min and a return to baseline values at 240 min. Similar postprandial kinetics of LDL-C and HDL-C have been observed in large cohort studies [36], men with metabolic syndrome [37] and patients with abdominal obesity [38]. The LDL-C reduction could be attributed to increased intake of LDL-C by peripheral tissues while the reduction in HDL-C is a result of the bidirectional transfer of TGs from TG-rich lipoproteins (TRLs) to HDL and to a concomitant transfer of cholesterol ester from HDL to TRLs through the action of cholesterol ester transfer protein (CETP) [39]. The OO500 meal seems to induce the highest decline of LDL-C compared to other meals but this effect reached statistical significance only between OO250 and OO500. Oleocanthal is able to increase the expression and activity of the LDL receptor-related protein 1 (LRP1) in cultured mice brain endothelial cells. LRP1 belongs to the family of LDL receptors and a possible activation of LDLR by oleocanthal could partly explain the highest postprandial decrements of LDL-C due to increased clearance of LDL-C. However, there are no experimental studies for this putative mechanism [40].

We also decided to study the postprandial kinetics of uric acid [41] and homocysteine [42,43] as both factors are putative activators of platelets and their levels are augmented in diabetic patients [44,45]. According to our results, all meals induced a slight decrease in uric acid from baseline levels 3 h after the consumption of all meals. The postprandial decline of uric acid is similar between meals. A transient, albeit non-significant, reduction in homocysteine was observed 90 min after the consumption of all meals. The kinetics of homocysteine were similar for all meals. A similar decline of uric acid levels 2 h after a glucose challenge was observed by Tinahones et al. [46], who attributed this decline to the consumption of uric acid in redox reactions trying to face the increased postprandial oxidative stress. Whether this is true for our study remains to be elucidated when postprandial markers of oxidative stress will be determined. Nevertheless, the similar kinetics of all the aforementioned indices of glycemic control and lipid metabolism after all meals indicates that they elicited similar redox responses in the participants. 

A trend for a decline of homocysteine levels is observed after the consumption of all meals (7–11%) which reached significant levels at 90 min for BU, BU-IBU and OO and at 240 min for BU, OO and OO500. A similar acute decline of homocysteine was observed after the consumption of a mixed meal in both younger and older adults [47]. The micronutrient and amino acid profile of a mixed meal provide metabolites and amino acids such as choline, betaine and folic acid that may acutely affect postprandial homocysteine degradation. However, our light and relatively simple test meal is unlikely to affect homocysteine metabolism through its micronutrient content, as was observed in adolescents consuming a vitamin fortified beverage 40 min before an OGTT [48]. It seems that insulin secretion drives the postprandial decline of homocysteine since physiological hyperinsulinemia can stimulate homocysteine metabolic clearance [49]. If this is the case then the non-significant time x intervention effect of meals on postprandial homocysteine follows a similar pattern observed for postprandial hyperinsulinemia. 

The main aim of this study was to assess the acute antiplatelet properties of oleocanthal-rich olive oils in comparison to oleocanthal-poor olive oil, butter and butter plus ibuprofen as a positive control. Oleocanthal is a COX1,2 inhibitor [18] and through the attenuation of TxA2 production by the activated platelets may exert antiplatelet properties. It is therefore obvious that the consumption of oleocanthal-rich olive oils may induce an acute antiplatelet effect, which has already been demonstrated in healthy subjects [20]. However, we wanted to investigate this notion under real postprandial conditions in diabetic patients for several reasons. Firstly, because the consumption of EVOOs is usually achieved in the context of mixed meals with macro- and micronutrient profiles that may affect or mask the antiplatelet properties of oleocanthal-rich olive oil. Secondly, the postprandial hyperglycemia and hyperinsulinemia, which is often observed in diabetic patients, may activate platelets, although this phenomenon depends on many factors including type of diabetes and composition of the meal. The daily activation of diabetic platelets by postprandial dysmetabolism may be a chronic stimulus of atherosclerotic processes induced by the subclinical activation of platelets. Therefore, our hypothesis was that the consumption of oleocanthal-rich olive oils could counteract the platelet activation induced by the postprandial hyperglycemia/hyperinsulinemia in a dose-dependent fashion and comparably to ibuprofen. The outcomes of our study have shown that the control meals (BU and OO), which did not contain anti-COX molecules (either ibuprofen or oleocanthal), could not affect the ex vivo activation of platelets by ADP or TRAP. Previous studies, which investigated the effect of hyperglycemia/hyperlipidemia after glucose or fat loads, provided conflicting results on the ability of these loads to activate diabetic platelets. Yngen et al. have shown that glucose loads elevated soluble P-selectin in males with mild type II diabetes mellitus but decreased ADP- and thrombin-induced platelet P-selectin expression [50]. On the other hand several studies have shown that glucose [51,52] or fat loads [53] could not alter or even reduce platelet aggregability or activation in insulin resistant and/or diabetic patients. Mixed meals of higher caloric content seem to have a stronger impact of postprandial platelet functionality. A standardized meal was able to enhance ADP-induced platelet P-selectin expression in Type 2 diabetic patients [54], while the same group from Karolinska has shown that a mixed meal also increased a U46619-induced platelet P-selectin expression, fibrinogen binding and platelets-leukocytes aggregates formation of platelet-derived microparticles 90 min after the meal [22,23,55]. More importantly, the same group has shown that postprandial platelet activation correlated positively to postprandial insulin levels and inversely to glucose levels [55]. It is therefore obvious that the effect of the postprandial state on the activation and functionality of diabetic platelets is less than clear and is dependent on many factors such as the stimulus (oral/fat load, mixed meal), the state of insulin resistance and the assays utilized for the assessment of platelet reactivity. Several molecular mechanisms, activated under postprandial conditions, may have opposite effects on platelets’ functions, and the final phenotypic changes seem to be the net result of those mechanisms. For example, hyperglycemia/hyperlipidemia-induced oxidative stress and inflammation may activate platelets either directly or by impairing NO bioavailability [56,57] while at the same time insulin can exert mild antiplatelet effects, at least in insulin sensitive subjects [58,59,60]. Our control meals (BU and OO) induced a similar mild hyperglycemia/hyperinsulinemia which probably could not significantly affect molecular mechanisms of platelet activation. They differed only in the source of fat and the fatty acid content of the meal (SFA vs. MUFA). Previous studies comparing the fatty acid composition of the meals on in vitro [61] or ex vivo platelet aggregation [62] demonstrated that the effect of the meals on platelet activation are not dependent on the fatty acid content of the meals, similarly to our study. 

The primary hypothesis of our study was that oleocanthal-rich olive oils could exert acute antiplatelet properties, based on the COX inhibitory properties of oleocanthal [18] and previous positive experiments with polyphenol-rich olive oils in healthy volunteers [20]. Our hypothesis was confirmed since OO250 and OO500 meals demonstrated a dose-dependent and sustained antiplatelet effect between 90 and 240 min. The concentration of both ADP and TRAP, able to induce 50% of the maximum ex vivo aggregation (EC_50_), increased in a dose- and time-dependent manner, indicating the attenuation of platelet sensitivity to the aggregating agents. The antiplatelet effect of the oleocanthal-rich olive oil is comparable to that achieved by the administration of a 400 mg ibuprofen pill half an hour before the butter meal (BU-IBU), demonstrating the efficiency of the oleocanthal-rich olive oils to acutely attenuate platelet aggregation. The kinetics of EC_50_ changes are similar for both aggregating agents. An acute increase in EC_50_ at 90 min was observed after BU-IBU and OO500. The EC_50_ remained elevated until 4 h after the meals. The OO250 induced a more linear increase in EC_50_ values which were at any time lower than the EC_50_ values observed after BU-IBU and OO500. Our study shows that the in vitro antiplatelet properties of oleocanthal are confirmed in a real postprandial setting and at olive oil doses that are achievable during a meal. Similar studies with ours have shown that other polyphenol-rich foods, such edible wild plants and wine, could reduce postprandial platelet sensitivity to various aggregating agents in metabolic syndrome patients [63] and healthy volunteers [64], respectively. We conducted a pooled correlation analysis between iAUCs in order to assess whether the postprandial kinetics of the metabolic markers correlates with the postprandial responses of EC50 but no significant correlation was found. We also calculated the Δ[iAUCBU-IBU-iAUCBU] and Δ[iAUCOO500-iAUCOO] and made a correlation analysis to assess if the response of platelets to the ibuprofen is analogous to the respective response to oleocanthal, but again no significant correlation was found. We therefore believe that the antiplatelet effect of OO250/500 is independent of their ability to alter the kinetics of the metabolic markers and has to do with a direct effect of oleocanthal or its metabolites on circulating platelets. The usage of oleocanthal as an alternative monotherapy instead of classic NSAID could be proven efficient in the future; however, clinical trials are needed. Until then, olive oils rich in oleocanthal could serve as daily natural sources of oleocanthal, exerting mild antiplatelet actions. 

The current study has certain limitations. The size of the population was small, thus this study should be considered as a pilot study, and in no way may these results be generalized to the diabetic patients as a whole. The medication treatment of our volunteers was heterogenous and we cannot exclude the possibility that their medication could differentially affect their responses to the meals. The complete assessment of the postprandial hypertriglyceridemia required a longer follow up of at least 6 h. A wider panel of platelet functionality could be measured, such as P-selectin expression on platelet membranes or other soluble markers of platelet activity such as beta-thromboglobulin or platelet factor 4. On the other hand, this study, although preliminary has several strengths including its crossover design, the control of the dietary intake before the trials, the wide panel of metabolic markers which could affect platelet reactivity, the positive control meal of ibuprofen, the two dosages of oleocanthal-rich olive oils which revealed the dose-dependent effect of the phenomenon (at least between 250 and 500 mg/Kg olive oil) and the fact that the assessment of platelet reactivity was made at two different time point which demonstrated the sustainability of the antiplatelet activity for at least 4 h after the consumption of the meals.

## 4. Materials and Methods

### 4.1. Study Design

This is an acute, single-blinded, postprandial, study with a randomized crossover design. Patients with T2DM consumed 5 meals on 5 different mornings in a random order. All meals contained 120 g white bread combined with either: (a) 40 mL OO, (b) 40 mL OO250, (c) 40 mL OO500, (d) 39 g BU or (e) 39 g BU-IBU administered half an hour before the consumption of the meal. Blood samples were collected before and at 30, 60, 90, 120, 180 and 240 min after the meals. The primary aim of this study was to compare the acute effect of the different meals to the ex vivo sensitivity of platelets to two endogenous platelet activators, namely ADP and TRAP, while secondary outcomes were the effects of the meals on postprandial glycemia and lipemia. All procedures were in accordance with the Declaration of Helsinki and approved by the Ethics Committee of Harokopio University of Athens (ref. number 79548/16-05-2019). All participants signed a written informed consent before entering in the research procedure. The study was registered to ClinicalTrials.gov (ClinicalTrials.gov Identifier: NCT04419948). 

### 4.2. Participants

Eligible participants were non-insulin dependent adult patients (both males and females) with T2DM, whose diagnosis has been established by an endocrinologist or pathologist according to American Diabetes Association (ADA) criteria [18]. Their weight had to be stable (±3 Kg) for the last two months. There were no restrictions regarding smoking or the presence of menopause for the females. Exclusion criteria were anticoagulation or insulin therapy, diagnosis of chronic inflammatory disease, auto-immune diseases, cancer, uncontrolled thyroid disease, and consumption of food supplements in the last two months. Potential participants were informed about the study through word of mouth, social media, and an appropriately designed poster in collaboration with clinicians. Thirty patients were interested in participating in the study and were initially informed about it. Ten patients were under insulin therapy and five under anticoagulation therapy, so were not able to participate due to the exclusion criteria. From the remaining 15 volunteers with T2DM who started as participants of the study, 5 dropped out, and 10 volunteers (5 males and 5 females) completed the study. The medication of the participants is depicted in Table 1 of the Supplementary File (Table S1). All participants consumed Biguanide and 6 of them consumed HMG-CoA-reductase inhibitor, while there were no patients consuming fibrates, which may be an adjunct therapy for the case of the uncontrolled LDL [65].

### 4.3. Intervention Protocol

Participants visited the Metabolism Unit of the Department of Nutrition and Dietetics of Harokopio University of Athens six times. During their first visit, the volunteers were informed about the purposes and the procedures of the study and we collected demographic data and data for the medical history, medication, supplement consumption, anthropometry and nutritional and physical activity habits of the volunteers. If the participants fulfilled the inclusion criteria, they consumed the five meals on 5 different mornings in a random order. The order of the meals for each participant was achieved by the aid of a randomization software (Research Randomizer, randomizer.org). The meals were consumed at least two weeks apart for all participants. The volunteers came to the laboratory after 12 h overnight fast. They were given detailed guidelines to avoid the consumption of phenolic-rich foods and intense exercise three days before the intervention. Specifically, a registered dietician advised them to follow a diet with an approximate macronutrient composition of 55% carbohydrates, 17% protein and 28% fat. They were also advised to consume the same meal in the evening before each intervention. The volunteers were provided with three meal choices to choose. The dietary intake of the volunteers was assessed by three 24 h recalls the week before each intervention.

Before each meal, the volunteers rested for 15 min and blood pressure was measured while anthropometric data and pre-meal blood samples were collected. The meals were prepared on the day of the experiment. The participants were advised to consume the meals in a period of ten minutes, then they remained in the laboratory until the end of the experiment, avoiding intense physical activity and movements. Blood samples were also collected at 30, 60, 90, 120, 180 and 240 min after the initiation of the meal consumption. In the case of the BU-IBU meal, a pill of 400 mg ibuprofen (Nurofen) was administered to the volunteers after the pre-meal measurements and half an hour before the initiation of the meal. 

### 4.4. The Meals 

The meals of the study consisted of 120 g white bread (approximately 5 slices), combined with either 40 mL olive oil poor in phenolic compounds (meals OO, OO250, OO500) or 39 g butter (meals BU, BU-IBU). The bread was “Karamolegos Psichatost” which is a crustless wheat bread while the butter was “Lurpak^®^ Unsalted Butter”. The five different meals had similar caloric content and macronutrient composition, as seen in Table 2 of Supplementary file (Table S2). 

#### Olive Oil Characteristics and Production

For the study, we used three types of EVOO (OO500, OO250, OO). The OO500 was produced by olives (*Olea europaea* L.) of Kalamon variety harvested in November with malaxation for 45 min at 30 °C in the facilities of the OMPHAX SA olive mill. The olive oil was analyzed by qNMR to measure the phenol content as previously described [66]. The specific oil was found to contain oleocanthal 500 mg/Kg while all the other common phenolic compounds (oleacein, oleuropein aglycon, ligstroside aglycon, oleokoronal, oleomissional, tyrosol) were <10 mg/Kg. The oil was examined for its acidity (0.3%), peroxide value (5 meq O_2_) and K indexes (K_232_ = 1.851, K_270_ = 0.19), and was found to comply with the extra virgin category.

The OO was prepared after mixing of OO500 with an equal volume of water (30 °C) and mechanical stirring for 30 min, then separation with centrifugation in the facilities of the OMPHAX SA olive mill. This procedure was repeated three times until the oleocanthal content dropped < 10 mg/Kg measured by qNMR. The lipid profile of OO remained identical with that of OO500 as measured by qNMR. Oleocanthal is known to react with water to form a hydrosoluble 1,1-diol (oleocanthadiol) [67] that can be transferred from the olive oil layer to the aqueous layer during the centrifugation. The OO250 was produced by mixing equal amounts of OO500 and OO so the oleocanthal content was adjusted at 250 mg/Kg as measured by qNMR. The lipid profile of OO250 remained identical with that of OO500 as measured by qNMR.

### 4.5. Assessment of Dietary Intake 

The dietary intake of the volunteers before each meal was assessed by three 24 h dietary recalls and a semi-quantitative food frequency questionnaire (FFQ) collected the week before each trial. The 24 h dietary recalls were completed the day before the intervention, a random day of the week and a weekend day [68]. Nutritionist Pro™ software (version 2.2, Axxya Systems LLC, Stafford, TX, USA) was used for the analyses. The FFQ [69] was completed on participants’ first visit, and it was able to estimate the frequency of the consumption of the main food items and the MedDiet Score. All questionnaires were completed through an interview (face to face or by a phone call).

### 4.6. Anthropometry 

Anthropometric characteristics were measured on each visit, with participants in light clothing and without shoes. A digital scale for the weight (Seca 861; Seca, Vogel & Halke, Hamburg, Germany) and a stadiometer for height (Seca Leicester Height Measure; Seca, Vogel & Halke) were used. The measurements were made to the nearest 0.1 kg and 0.1 cm respectively. Body mass index was calculated by the formula BMI = Weight (Kg)/Height (m) × Height (m) [70]. Body composition was estimated by bioelectrical impedance analysis (BC-418 MA, Tanita Corp., Tokyo, Japan). Waist circumference was measured, with the tape being placed at the mid-axillary line, around the lateral aspect of each ilium [71]. Resting arterial blood pressure was measured three times in the left arm in a sitting and resting position [72] by the electronic device “Omron M6 Comfort”.

### 4.7. Blood Sample Collection 

Before the consumption of the meals, an intravenous catheter was placed in the brachial vein of the volunteers and a fasting, pre-meal blood sample was collected in proper vacutainers or plastic tubes for the isolation of serum, plasma and platelet-rich plasma. Post-meal blood samples were collected at 30, 60, 90, 120, 180 and 240 min. For the isolation of serum blood samples were collected in vacutainers without anticoagulant. Blood was allowed to clot at room temperature for 30 min until centrifugation (1500× *g* for 20 min at 4 °C). Plasma was isolated from EDTA anticoagulated whole blood by centrifugation at 1500× *g* for 10 min at 4 °C. All samples were immediately aliquoted in Eppendorf tubes and stored at −80 °C.

For the isolation of platelet-rich plasma, blood samples were collected at baseline and postprandially (t = 90 min and t = 240 min) to 15 mL Falcon tubes containing 1.4 mL of 3.2% *w*/*v* trisodium citrate as anticoagulant. Platelet-rich plasma (PRP) was obtained by centrifugation of citrated whole blood at 180× *g* for 10 min at room temperature. The centrifugation pellet was then centrifuged at 1500× *g* for 20 min at room temperature and the platelet-poor plasma (PPP) was also collected. 

### 4.8. Platelet-Rich Plasma Aggregation 

The ex vivo human platelet aggregation assessment has already been described [73]. PRP and PPP were obtained by the centrifugations mentioned above. The aggregation induced by various concentrations of ADP or TRAP was measured in PRP with the method of light transmittance aggregometry in a Chrono-Log (Havertown, PA, USA) aggregometer (model 440VS). An evaluation of the maximum reversible or the least not reversible platelet aggregation was evaluated for both ADP and TRAP in order to assess the 100% aggregation. During 20% to 80% of the maximum reversible platelet aggregation, a linear curve between the concentration of the agonist used and percentage of aggregation was formed. From this linear curve, the equivalent concentration for 50% aggregation (EC_50_) was calculated. Specifically, the concentration of each aggregatory agent (ADP, TRAP) that induces 50% of maximum aggregation was evaluated.

### 4.9. sP-Selectin

The soluble P-Selectin (sP-selectin) was measured in plasma with a commercially available ELISA kit (Human P-Selectin/CD62P DuoSet ELISA) at baseline and at 30, 60, 90, 120, 180 and 240 min after the consumption of the meals.

### 4.10. Metabolic Markers 

Peripheral blood samples were collected from the 10 participants for subsequent analysis. Glucose, insulin, c peptide, uric acid, blood lipid profile, markers of liver function and the acute inflammation marker C-reactive protein (CRP) were measured with the use of a biochemical analyzer (Atellica Solution Siemens). Homocysteine was measured with the use of an Immunochemistry analyzer (Centaurus XPT Siemens). All serum analyses were conducted at the same lab following the same procedure.

### 4.11. Statistical Analysis

Analysis was performed using SPSS v.24 (SPSS Inc., Chicago, IL, USA) and the significance level was set at 0.05. The normality of the variables was tested using the Kolmogorov–Smirnov test. Independent samples t-test or the Mann–Whitney U test were used for the comparisons of baseline values according to their distribution. Repeated measures ANOVA was used for the comparisons (ptime, pintervention, ptimexintervention) of normally distributed variables during each intervention. Paired samples *t*-test was used for the comparison of each time point to the baseline and be-tween interventions. The AUCs were calculated using the GraphPad Prism 8.4.3 software. The correlations between the variables were analyzed by Pearson’s or Spearman correlation for normally and non-normally distributed variables.

## 5. Conclusions

In conclusion, the consumption of 40 mL of olive oils enriched with oleocanthal reduced platelet sensitivity to ADP and TRAP at least 4 h after the consumption of the meals in a dose-dependent manner and independently of postprandial glycemia and lipidemia. The inclusion of meals which contain oleocanthal-rich olive oils in the dietary plan of diabetics may provide a daily, mild, more sustainable antiplatelet therapy to diabetic patients. Larger randomized controlled trials are required to prove this hypothesis.

## Figures and Tables

**Table 1:** Anthropometric, clinical characteristics and dietary intake before each meal.

Studied Parameter	Mean ± Std. Error					*p*-Value
	Butter (BU) (N = 10)	Butter and 400 mg Ibuprofen (BU-IBU) (N = 10)	Olive Oil Poor in Phenolic Compounds (OO) (N = 10)	OO Containing 250 mg/Kg Oleocanthal (OO250) (N = 10)	OO Containing 500 mg/Kg Oleocanthal (OO500) (N = 10)	
Age (years)	53.8 ± 1.3					
Systolic blood pressure (mmHg)	126 ± 4.2	123 ± 3.7	129 ± 3.2	126 ± 5.1	127 ± 5.5	0.937
Diastolic blood pressure (mmHg)	76 ± 3.3	74 ± 2.6	75 ± 1.7	71 ± 4.1	73.2 ± 3.4	0.824
Body Mass Index (kg/m^2^)	33.8 ± 2.2	33.7 ± 2.1	33.4 ± 2.2	33.8 ± 2.1	33.7 ± 2.2	0.995
Waist circumference (cm)	110.8 ± 3.7	110.0 ± 3.2	110.0 ± 3.7	110.5 ±3.5	110.7 ± 3.8	0.998
Body fat % (according to bioelectrical impedance analysis)	36.6 ± 5	38.8 ± 4.2	38.7 ± 3.9	40.0 ± 4.4	38.3 ± 4.3	0.988
Meditteranean Diet score	38.5 ± 0.9					
Energy intake (Kcal)	2352 ± 391	2363 ± 351	2143 ± 248	2487 ± 414	2608 ± 406	0.922
Fat (% of total energy intake)	45.6 ± 3.5	48.7 ± 3.4	49.2 ± 2.7	71.2 ± 17.3	46.1 ± 2.5	0.179
Carbohydrates (% of total energy intake)	40.2 ± 3.4	34.7 ± 4.2	41.3 ± 4.3	40.9 ± 2.6	38.8 ± 2.4	0.695
Protein (% of total energy intake)	13.1 ± 1.2	16.4 ± 1.4	15.1 ± 2	14.0 ± 1.2	14.5 ± 0.9	0.573
Saturated fatty acids, SFA (g)	32.1 ± 4.6	33.1 ± 4.3	28.7 ± 3.5	35.6 ± 4.0	40.6 ± 9.5	0.664
Monounsaturated fatty acids, MUFA (g)	49.7 ± 6.8	52.2 ± 6.3	55.1 ± 4.6	51.4 ± 3	53.5 ± 5.3	0.960
Polyunsaturated fatty acids, PUFA (g)	9.5 ± 0.9	10.2 ± 0.8	11.9 ± 0.9	10.0 ± 1.0	19.7 ± 1.2	0.738
Omega-3 fatty acids (g)	0.8 ± 0.1	0.8 ± 1.9	1.0 ± 0.4	0.7 ± 0.4	0.8 ± 0.1	0.500
Omega6 fatty acids (g)	6.6 ± 0.6	7.1 ± 0.6	7.2 ± 0.8	6.7 ± 0.5	7.6 ± 0.8	0.872

**Table 2:** Basic biochemical markers before each meal.

Studied Parameter	Mean ± Std. Error					*p*-Value
	BU (N = 10)	BU-IBU (N = 10)	OO (N = 10)	OO250 (N = 10)	OO500 (N = 10)	
Glucose (mg/dL)	111 ± 6.0	110 ± 6.0	109 ± 5.3	114 ± 6.8	109 ± 6.6	0.985
Insulin (mU/L)	15.2 ± 3.3	15.9 ± 3.8	16.9 ± 5.4	15.2 ± 2.7	15.4 ± 3.6	0.998
HOMA-IR	4.2 ± 1.0	4.4 ± 1.2	4.4 ± 1.3	4.3 ± 0.8	4.4 ± 1.3	0.995
C peptide (ng/mL)	2.3 ± 0.3	2.3 ± 0.3	2.4 ± 0.4	2.4 ± 0.2	2.3 ± 0.3	0.993
Cholesterol (mg/dL)	177 ± 13	174 ± 13	174 ±15	171 ± 15	174 ± 11	0.999
HDL (mg/dL)	46.5 ± 4.6	45.4 ± 3.6	44.9 ± 3.4	46.8 ± 4.6	46.9 ± 3.4	0.995
LDL (mg/dL)	122 ± 16	122 ± 14	124 ± 18	121 ± 16	120 ± 13	0.993
Triglycerides (mg/dL)	168 ± 27	144 ± 14	125 ± 15	132 ± 17	152 ± 20	0.570
Uric Acid (mg/dL)	5.4 ± 0.5	5.6 ± 0.5	5.5 ± 0.5	5.6 ± 0.5	5.5 ± 0.5	0.996
GGT (U/L)	32 ± 7	30 ± 7	31 ± 5	31 ± 7	31 ± 8	0.998
CRP (mg/dL)	4.2 ± 1.4	3.4 ± 1.5	3.7 ± 1.5	3.9 ± 1.4	3.7 ± 1.2	0.999
Homocysteine (mg/dL)	14.2 ± 1.0	13.6 ± 1.4	14.8 ± 1.1	14.1 ± 1.1	15.0 ± 1.4	0.938

## Data Availability

Data are unavailable due to privacy.

## Supplementary Materials

The supporting information can be downloaded at: https://www.mdpi.com/article/10.3390/ijms25020908/s1.
