# Supplementary material for: Acute Antiplatelet Effects of an Oleocanthal-Rich Olive Oil in Type II Diabetic Patients: A Postprandial Study
Source: Int J Mol Sci. 2024 Jan 11;25(2):908. doi: 10.3390/ijms25020908 (PMC10815739; doi:10.3390/ijms25020908)
Supplement: Supplementary file 1 [file ijms-25-00908-s001.zip › ijms-2780487-supplementary.pdf]

Table S1. Participants' medication.

	Volunteer code: 1	Volunteer code: 2	Volunteer code: 3	Volunteer code: 4	Volunteer code: 5	Volunteer code: 6	Volunteer code: 7	Volunteer code:8	Volunteer code:9	Volunteer code: 10
**Biguanide**	√	√	√	√	√	√	√	√	√	√
**Thiazolidinediones**	√									
**GLP-1 analogues**					√					
**HMG-CoA-reductase inhibitor**		√	√	√	√	√		√		
**Proton pump inhibitor**					√					
**ACE inhibitor**		√			√	√				
**B blocker**		√			√					
**Calcium channel blockers**		√				√				
**SGLT2 inhibitors**								√		
**DPP-4 enzyme inhibitor**						√				

Table S2. Five different meals' caloric content and macronutrient composition.

	BU	BU-IBU	OO	OO250	OO500
**Energy (Kcal)**	695	695	687	687	687
**CHO (g)**	56	56	54.2	54.2	54.2
**Fat (g)**	45	45	45	45	45
**Protein (g)**	16.6	16.6	16.3	16.3	16.4
**CHO (%)**	32.2	32.2	31.5	31.5	31.5
**Fat (%)**	58.3	58.3	58.9	58.9	58.9
**Protein (%)**	9.5	9.5	9.6	9.6	9.6
